# Nutritional Management of Very Sick Patients: Paradigm Changes and Needs for Further Research

**DOI:** 10.1016/j.advnut.2024.100348

**Published:** 2024-11-26

**Authors:** Steven A Abrams, Jaclyn L Albin

**Affiliations:** 1 Department of Pediatrics, Dell Medical School at the University of Texas at Austin, Austin, TX, United States; 2 Department of Internal Medicine and Pediatrics, the University of Texas Southwestern Medical Center, Dallas, TX, United States

In this issue of Advances in Nutrition: An International Review Journal, we are publishing 2 articles addressing the nutritional needs of hospitalized adults. One article by Ramaswamy et al. [1] discusses critically ill adults, whereas Heutlinger et al. [2] discusses adults who are undergoing or recovering from a major surgical intervention.

These articles provide a clear description of the need to change both many of our long-held biases in these areas and to conduct further, well-designed studies that focus on these highly vulnerable populations and how best to provide evidence-based nutritional care for them. It is a major step forward to openly discuss issues that often were governed by a perception that critically ill or operative patients had greater concerns than adequate nutrition, and thus nutrition could largely be ignored for an extended period of time. Days of hanging bags of intravenous fluids without protein or critical micronutrients and long delays in enterally feeding patients need to disappear and be replaced by comprehensive, mandatory multidisciplinary assessments of nutritional status and consideration of the value of healthy nutrition in recovery and long-term outcomes in hospitalized patients.

In reviewing the article by Ramaswamy et al. [1], as a neonatologist and a pediatrician, we were struck by the 9 myths related to adult critical care and how every single one, with some minor semantic tweaking, could be applied to critically ill neonates and small children! Certainly, growth and neurocognitive development are more central themes in neonates and small children than in adults, but the value of using the intestine to feed whenever possible and finding meaningful strategies for feeding operative or critically ill patients remains very much the same across the lifespan. In particular, Myth 4 about gastric residuals having a very limited value in determining nutritional strategy [3], and Myth 8 about vasopressor use being consistent with permitting enteral nutrition in the appropriate clinical situation, especially strike home in thinking about the similarities of caring for ill neonates just as for adults [4]. Good clinical judgment remains paramount, including recognition of limitations involved in feeding in these situations in all ages, but removing absolutes based on historical biases that were not data-supported is a huge step toward improving short and long-term patient outcomes.

Moving forward, these articles make it very clear that we have a compelling need to conduct more research into the ideal nutritional strategy for care of critically ill and surgical patients, including the development of broad, multidisciplinary approaches for nutritional management of hospitalized individuals of all ages. We note this comment from Heutlinger et al. [2]: “Given the low risk, high reward nature of perioperative nutritional intervention and the large sector of patients who fail to be identified as malnourished during the operative period, the development of dietitian driven clinical follow-up for patients to standardize nutritional optimization across the spectrum of surgical care may be a beneficial step for future care.*”* This is absolutely the case! Given the authors’ concomitant recognition that nearly half of surgical patients suffer from some degree of malnourishment, which is likely an underestimate, especially given historical emphasis on defining this as low BMI when also obesity often reflects a state of malnourishment [5], what if pre- and postsurgical nutrition care became ubiquitous? Indeed, such a high-yield intervention to promote nutritional care guidelines for all surgical patients could positively impact surgical outcomes.

Physicians need to be aware of the importance of consistent plans and strategies for nutritional management, but they must also have available fully funded and well-trained dietitians and other team members, such as pharmacists, to support them in daily patient management and guideline creation. Nutrition education for physicians is critical [6] and can guide physicians and dietitians, along with others in the health care team, in developing systematic strategies for nutritional support of patients of all ages facing critical illnesses, surgery, and recovery. In fact, opportunities to emphasize the vital role of nutrition in health particularly emerge during the vulnerable, life-changing experiences of critical illness and surgery.

Overall, these articles provide the opportunity to seriously consider next steps, including how we can better provide research data to support innovative approaches to nutritional support for hospitalized patients. We welcome further articles on these topics and ongoing discussion of the evidence for proposed protocols and nutritional strategies.

## Author contributions

Both authors read and approved the final manuscript.

## Funding

The authors reported no funding received for this study.

## Conflicts of interest

The authors report no conflicts of interest.

## References

[1] Ramaswamy T., DeWane M.P., Dashti H.S., Lau M., Wischmeyer P.E., Nagrebetsky A. (2024). Nine myths about enteral feeding in critically Ill adults: an expert perspective. Adv. Nutr..

[2] Heutlinger O., Acharya N., Tedesco A., Ramesh A., Smith B., Nguyen N.T. (2024). Nutritional optimization of the surgical patient: a narrative review. Adv. Nutr..

[3] Abiramalatha T., Thanigainathan S., Ninan B. (2019). Routine monitoring of gastric residual for prevention of necrotising enterocolitis in preterm infants. Cochrane Database Syst. Rev..

[4] Moliner-Calderón E., Verd S., Leiva A., Ginovart G., Moll-McCarthy P., Figueras-Aloy J. (2023). The role of human milk feeds on inotrope use in newborn infants with sepsis. Front. Pediatr..

[5] Freeman A., Aggarwal M. (2020). Malnutrition in the obese: commonly overlooked but with serious consequences. J. Am. Coll. Cardiol..

[6] Albin J.L., Thomas O.W., Marvasti F.F., Reilly J.M. (2024). There and back again: a forty-year perspective on physician nutrition education. Adv. Nutr..

